# Effects of visual feedback balance training with the Pro-kin system on walking and self-care abilities in stroke patients

**DOI:** 10.1097/MD.0000000000022425

**Published:** 2020-09-25

**Authors:** Min Zhang, Hong You, Hongxia Zhang, Weijing Zhao, Tingting Han, Jia Liu, Shangrong Jiang, Xianhui Feng

**Affiliations:** Sino-French Department of Neurological Rehabilitation, Gansu Provincial Hospital, Lanzhou, Gansu, China.

**Keywords:** balance training, Pro-kin system, self-care abilities, stroke, visual feedback, walking

## Abstract

Some scholars’ studies have demonstrated that Pro-kin balance system training is able to promote the recovery of the balance function in stroke patients. The present study has expanded on those studies, and was not merely limited to studying balance, but also encompassed walking and self-care abilities of the patients; furthermore, the association among balance and walking and self-care abilities was also explored.

A total of 40 stroke patients were randomly and equally divided into 2 groups: the control group (n = 20) and the treatment group (n = 20). Both groups underwent conventional balance training, although the treatment group also underwent visual feedback balance training with the Pro-kin system. The balance function was assessed using the Berg Balance Scale (BBS), the Timed “Up & Go” (TUG) test, and Pro-kin system parameters. The Pro-kin system parameters included the perimeter and ellipse area, which were both tested once with eyes open (EO) and eyes closed (EC). Walking ability was assessed using the Holden Walking Ability Scale, according to the Functional Ambulation Classification (FAC). The self-care abilities were assessed with the Barthel Index (BI). The tests were conducted prior to training, and 3 weeks after the end of the training programme.

No significant differences were noted among the groups before the training. After 3 weeks of training, for both the groups, significant improvements in balance and the walking and self-care abilities were noted: The BBS value was significantly increased (*P* < .05), whereas the TUG, perimeter, and ellipse area with EO and EC measurements were significantly decreased after treatment (*P* < .05). The FAC and BI readings were significantly increased after treatment (*P* < 0.05), and the treatment group outperformed the control group (*P* < .05). Furthermore, the balance function was shown to be strongly correlated with the walking and self-care abilities (*P* < .01).

The present study has demonstrated that the use of the Pro-kin visual feedback balance training system in combination with conventional training is a viable method for improving walking and self-care abilities of stroke patients.

## 1 Introduction

**Figure 1:**
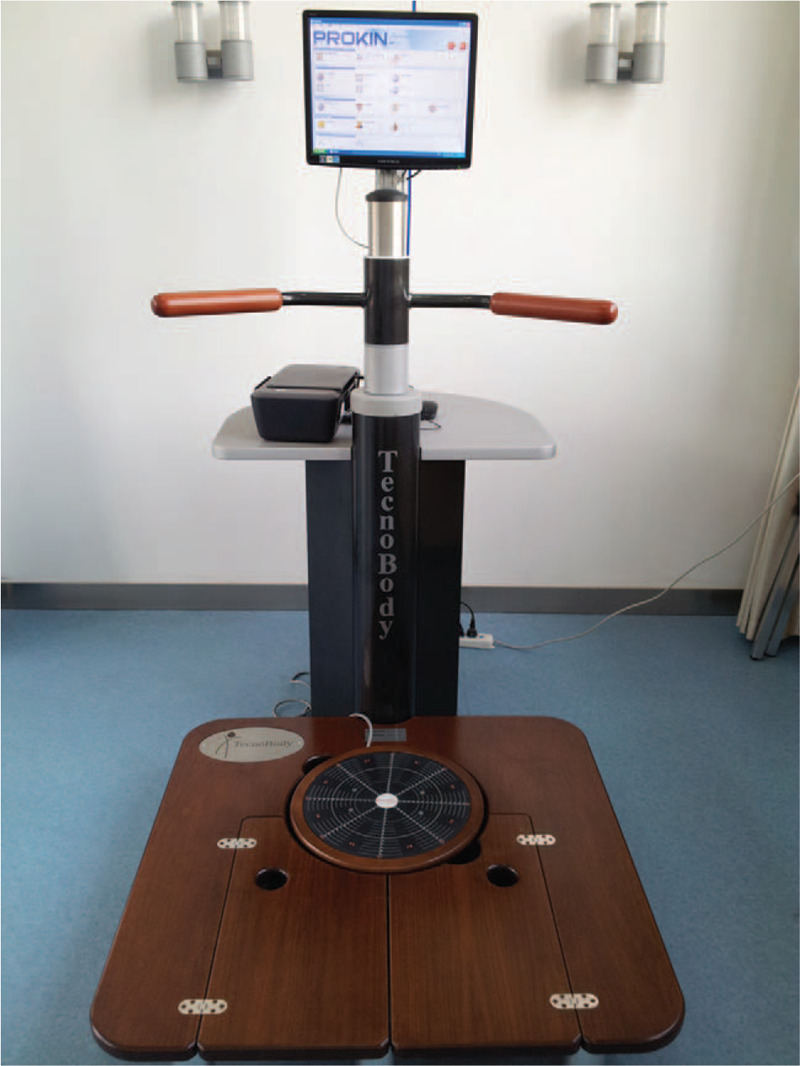
Pro-kin system.

Stroke is one of the most common causes of human death and disability.^[1,2]^ Balance problems are a common dysfunction that arises after stroke, and they have been implicated in poor recovery of both standing and walking ability of the patients, as well as an increased risk of falls.^[3–6]^ How to improve the balance function in stroke patients has become a hot topic in neurological rehabilitation research. Various therapeutic methods, including core-strength exercises,^[7]^ visual feedback training,^[8]^ and task-related training,^[9]^ have been used to improve balance. Among the therapeutic methods used for this purpose, visual feedback training has been shown to be especially effective.^[10–13]^ Previous studies^[14–17]^ have highlighted the Pro-kin system (Fig. 1), a new type of commercially available computerized balance assessment and training system that provides the user with accurate visual feedback information about the position of the center of pressure (CoP). Liu^[14]^ demonstrated that Pro-kin balance instrument training can promote the recovery of the balance function of stroke patients; furthermore, Li et al^[15]^ reported that treatment with the Pro-kin balance system improved balance ability in stroke patients with hemiplegia.

The present study has expanded on those previous studies, not merely being limited to a study of the balance, but also encompassing walking and self-care abilities. Therefore, the present study has investigated the effects of visual feedback balance training with the Pro-kin system on walking and self-care abilities of stroke patients; furthermore, the associations among balance and the walking and self-care abilities in stroke patients were also explored.

## 2 Methods

### 2.1 Study participants

The study group included 40 participants (the minimum sample size that can satisfy the test efficiency is determined by using the sample size calculation formula of the superiority test) with a radiological diagnosis of stroke, who were patients at Gansu Provincial Hospital between November 2018 and December 2019. All the participants were between 50 and 80 years of age, and had no history of leg injuries or other diseases associated with balance impairments; they also scored <56 on the Berg Balance Scale (BBS) and >22 on the Mini-Mental State Examination (MMSE). Individuals were referred if they had experienced a stroke within the last 3 months, could stand unassisted for 1 minute, and needed balance training, according to the judgement of the senior physical therapist. Candidates were excluded if: they had recurrent strokes; they had bilateral hemispheric, cerebellar, or brain stem lesions; they experienced severe spasticity or cognitive deficits; they had orthopedic problems; they experienced peripheral neuropathy, significant visual-field or hemineglect problems; they had impairments of the cardiovascular or respiratory system; or other accompanying ailments were present. Individuals who participated in <80% of the exercise program, and those who were unable to perform follow-up tests, were also excluded from the final analyses.

The pool of participants was randomly and equally divided into a treatment group (n = 20) and a control group (n = 20) according to a random number distribution table. The present study was approved by the Ethics Committee of Gansu Provincial Hospital, and all the participants provided written informed consent. All methods were performed in accordance with the relevant guidelines and regulations.

### 2.2 Data collection

Balance function was calculated by using the BBS^[18]^ and Timed “Up & Go” (TUG)^[19]^ tests. The BBS, a clinical functional measurement of balance impairment, consists of 14 items of increasing difficulty, which is scored on a 5-point ordinal scale (0 to 4). The maximum possible score is 56, and higher scores indicate an improved balance. In the present study, the TUG was also performed to assess the subjects’ balance; this test records the time (in sec) required for subjects to stand up from a chair, walk 3 m, turn around, return to the chair, and sit down again. Shorter times indicate better balance. Self-care ability performance was assessed using the Barthel Index (BI),^[20]^ which includes eating, washing, dressing, defecating, and transferring between a bed and a chair, among other activities. Each item has a minimum score of 0 and a maximum score of up to 15 points, for a total score of 100 points, where higher scores indicate improved self-care ability. The Functional Ambulation Classification (FAC), as previously described,^[21]^ was used to evaluate walking ability; this scale is divided into 5 grades, where higher grades indicate an improved walking ability. Four tests were repeated twice, and the mean scores were recorded as the results.

The present study also used the Pro-kin system (PK254, TenoBody s.r.l. Bergamo, Italy) to assess balance. This system utilizes a force-sensitive platform to assess postural sway from movements of the CoP. Subjects stood comfortably in a standardized position on the platform; they were instructed to look at a screen surface positioned straight ahead and to keep their arms at their sides while standing in a normal forward-facing position, with their eyes focused on a stationary target. Each participant performed 2 standing tests lasting 30 seconds each: One test with eyes open (EO), and one with eyes closed (EC). In each of these conditions, the perimeter (in mm) and ellipse area (in mm^2^) were measured, for a total of 4 different outcome variables. The test was performed twice, and the mean score was recorded.

### 2.3 Procedures

All participants received conventional balance training, which consisted of 5 practice sessions lasting 20 minutes each, conducted 5 times a week for 3 weeks. The exercises were as follows: standing on 1 leg for 5 seconds; standing in front of the mirror and being pushed by the therapists from different directions; shifting one's weight forward, backward, sideward, and diagonally with EO and EC; passing balls to the therapist that had been arranged in a circle, and throwing and catching a ball; and walking in a straight line.

**Figure 2:**
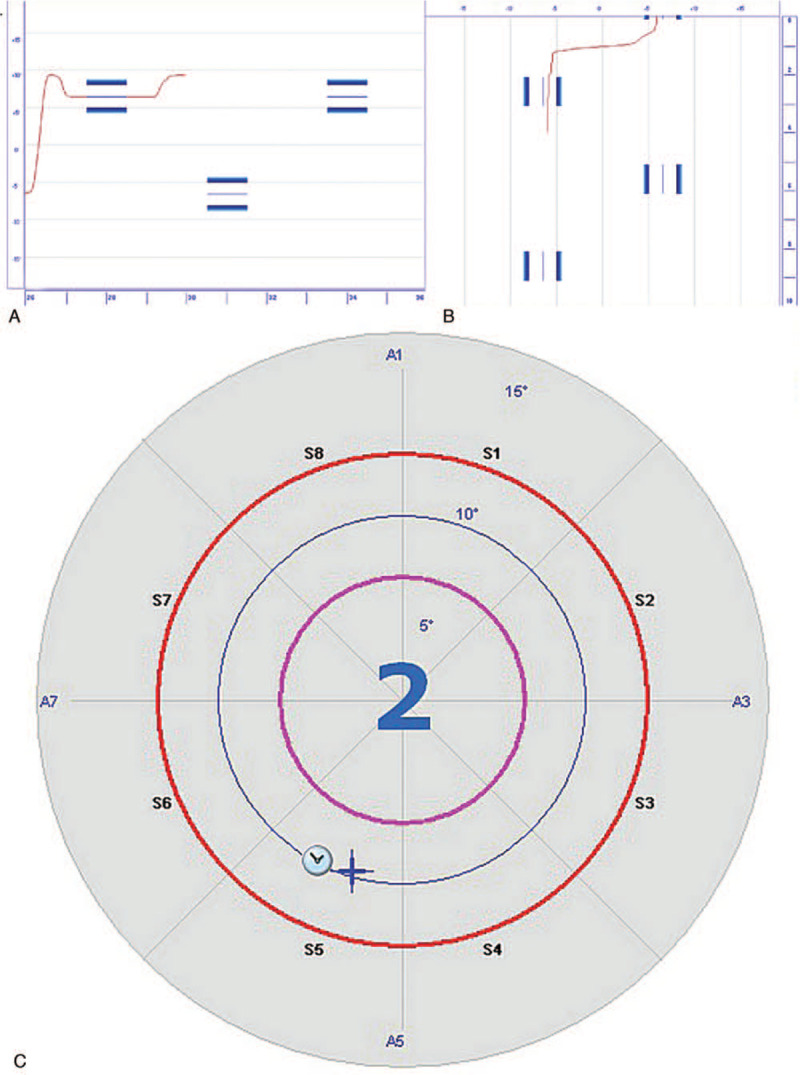
Modes of training using the Pro-kin system. (A) Forward and backward, (B) sideward, and (C) circular motion.

**Figure 3:**
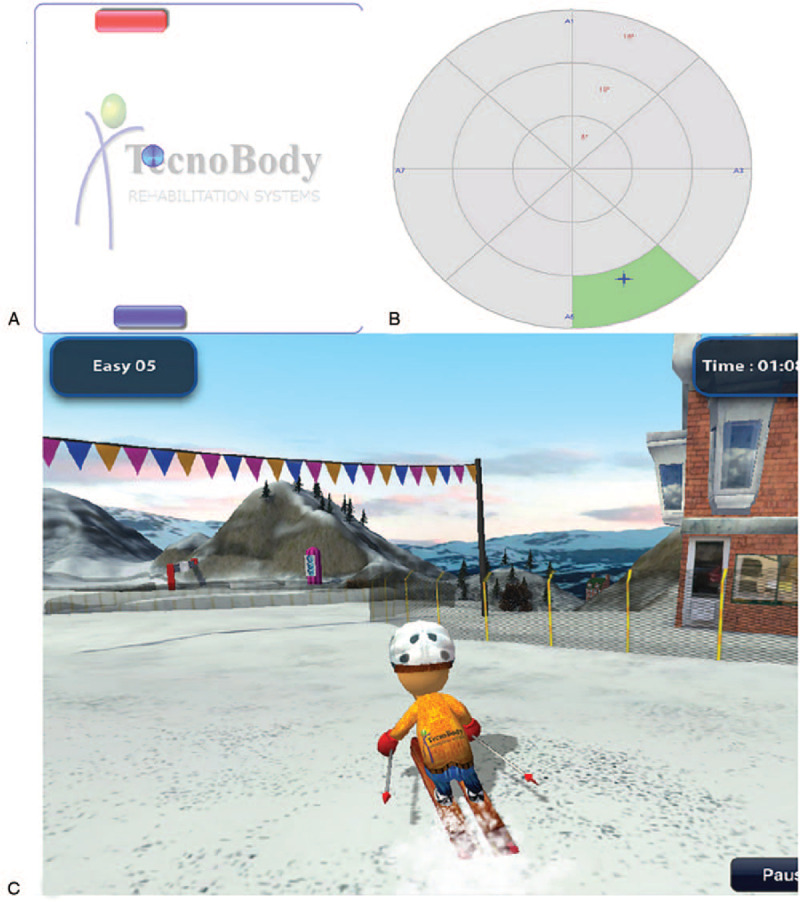
Game training. (A) Table tennis, (B) light, and (C) skiing.

All the subjects in the treatment group performed balance training using the Pro-kin system in addition to the conventional training; the subjects used the Pro-kin system for 20 minutes per session, 5 times a week, for 3 weeks. Using visual feedback sensitive to the displacement of the CoP, patients were required to move their CoP past the specified area in various directions, including forward, backward, sideward, and in a circular motion (Fig. 2). The patients also played 2 games of their choice, selected from 3 options: a table-tennis game, a light display, and a skiing simulator (Fig. 3).

### 2.4 Statistical analysis

All statistical tests were performed with SPSS version 17.0 (SPSS, Inc). Values were compared between groups using the independent *t* test, and values from before and after treatment were compared using the paired *t* test. Differences in categorical variables were analyzed using the chi-squared test. Correlations of balance function with walking and self-care abilities were determined by calculating Pearson correlation coefficient. For all tests, *P* < .05 was considered to indicate a statistically significant value.

## 3 Results

The general characteristics of the participants, including age, sex, height, weight, and lesion location, are described in Table 1. No significant differences were identified among the groups (*P* > .05). The BBS, TUG, FAC, and BI scores before and after training, along with the changes in those scores, are shown in Tables 2 and 3. The results of testing with the Pro-kin system before and after training, and the changes in those outcomes, are shown in Table 4. No significant differences were identified among groups before training; however, regarding the results before and after training in each group, the BBS, FAC, and BI values were significantly increased (*P* < .05), whereas the TUG, perimeter with EO, ellipse area with EO, perimeter with EC, and ellipse area with EC values were significantly decreased after training (*P* < .05), especially with the treatment group. The BBS, TUG, FAC, BI, and Pro-kin system results were significantly improved after training in the treatment group compared with the control group (*P* < .05).

**Table 1:**
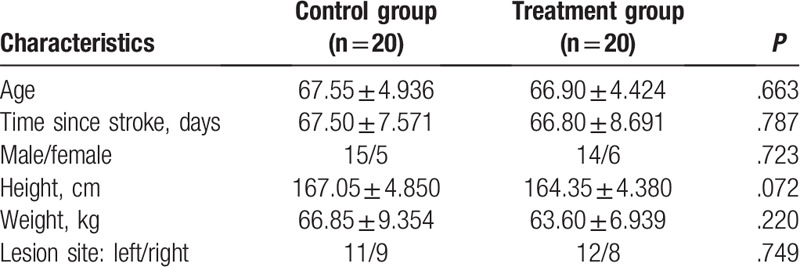
Characteristics of the participants.

**Table 2:**
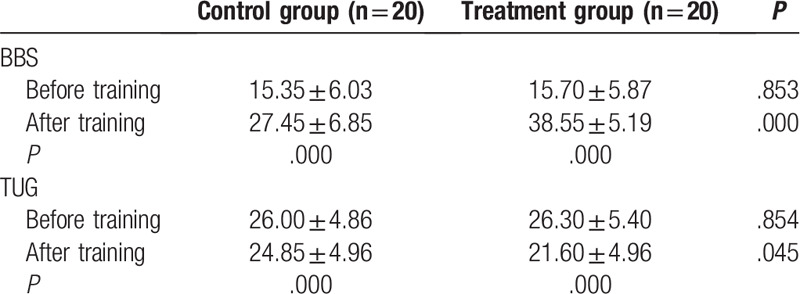
Comparison of BBS, TUG scores before and after training between the 2 groups.

**Table 3:**
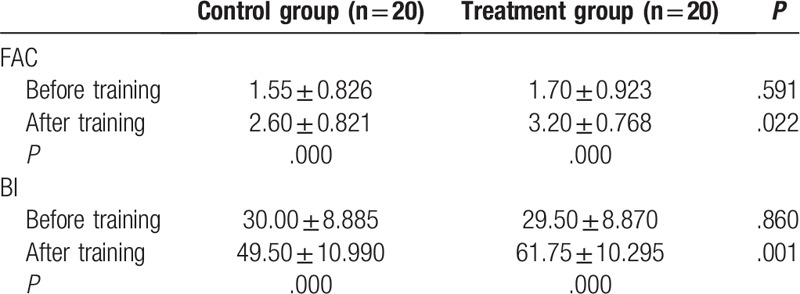
Comparison of FAC and BI scores before and after training between the 2 groups.

**Table 4:**
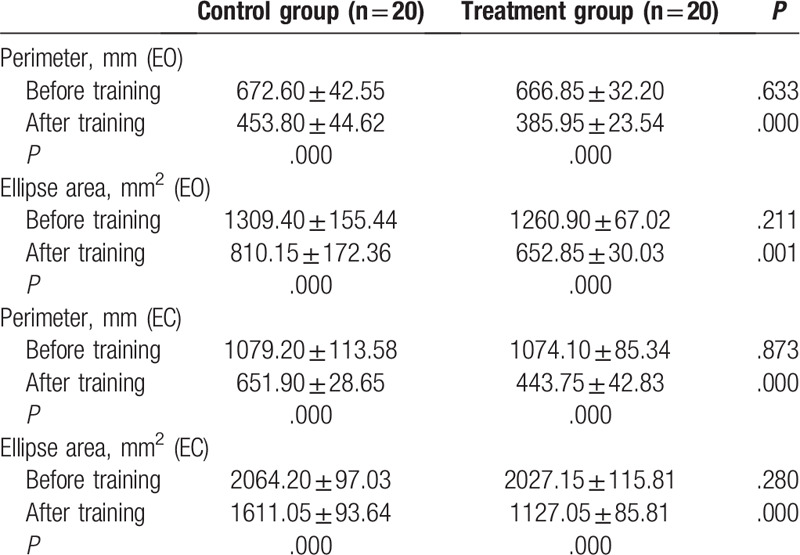
Comparison of perimeter and ellipse area before and after training between the 2 groups with eyes open and closed.

The correlations of balance function with walking and self-care abilities in the treatment group are shown in Table 5. The BBS and TUG values before training were significantly correlated with FAC (r = 0.934, *P* < .01; and r = −0.943, *P* < .01 for BBS and TUG, respectively) and BI (r = 0.973, *P* < .01; and r = −0.976, *P* < .01 for BBS and TUG, respectively) before training. The BBS and TUG values before training were also significantly correlated with FAC (r = 0.914, *P* < .01; and r = −0.918, *P* < .01 for BBS and TUG, respectively) and BI (r = 0.954, *P* < .01; and r = −0.972, *P* < .01 for BBS and TUG, respectively) after training. In order to evaluate the findings in the 2 groups further, the changes in all outcomes from baseline to posttraining were calculated; this demonstrated that there were clear changes in the treatment group for all outcomes.

**Table 5:**
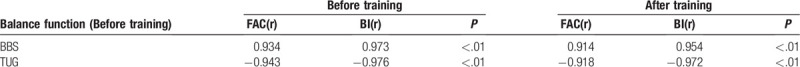
Correlation of balance function with walking and self-care ability (Treatment group).

## 4 Discussion

Stroke is often accompanied by deterioration or loss of balance function to varying degrees, and balance dysfunction is an important cause of stroke patients’ motor dysfunction. Therefore, training to recover balance function is crucial in the rehabilitation training of stroke patients.^[22–24]^

Previous studies have demonstrated that the balance function of the human body is controlled by the central nervous system through a variety of reflexes, adjustments based on peripheral proprioceptive and visual inputs, and the coordination of contractions among muscle groups, which comprise a series of complex processes.^[22,25]^ The proprioceptive hypofunction or vestibular paresthesia of stroke hemiplegic patients requires visual compensation for walking, flexion, and extension movements of affected limbs. Visual feedback associated with weight distribution and the position of the center of gravity was considered to improve the symmetrical posture of stroke patients, and this hypothesis has now been proven.^[26]^ For a long time, the Bobath technique and Brunnstrom method have been used as the main treatments for the balance function of stroke hemiplegic patients, with an emphasis placed on standing balance training and lower limb motion control training.^[27,28]^ However, these training methods are boring for the patients, their enthusiasm is restrained, and only modest improvements in balance are generally obtained. In recent years, visual feedback balance training with the Pro-kin system has gradually become an important treatment method. Through visual dynamic feedback with the Pro-kin system, patients are able to effectively control the movement of the body center of gravity and correct abnormal posture in time, thus improving the ability of patients to move the body center of gravity within the stable limit, and also improving their balance control function.^[14,15,29,30]^

The present study has amply shown that combined balance training using both conventional treatment and the Pro-kin system with visual feedback elicited significant improvements, outperforming conventional balance training alone in enhancing the balance function, walking and self-care abilities of our stroke patients. In a comparison of the results from before and after training, the BBS and the TUG outcomes were more dramatically changed after 3 weeks of training in both groups. Compared with the control group, the group that received conventional balance training and Pro-kin training also showed a significantly improved balance function. Pro-kin system test results were also significantly improved after 3 weeks of balance training. During the training process, whenever a patient's weight or posture shifts, the position and movement tracks of the center of gravity can be monitored; this information is presented through visual feedback, enabling a patient to adopt appropriate strategies to keep his or her posture as steady as possible,^[31]^ thus improving their balance ability. Our results are consistent with those of previous studies that have used the Pro-kin system in stroke patients,^[14,15,29,30]^ providing supporting evidence that using visual feedback from the force-sensitive platform is effective in reducing balance dysfunction in stroke patients. Moreover, in the present study, the perimeter and ellipse areas with EO were lower than with EC, since visual information can compensate for the loss of vestibular and proprioceptive function and facilitate the motor programs in the human brain, thus increasing the effectiveness of treatment.^[32–34]^

The walking and self-care abilities were also improved significantly for patients in the treatment group compared with those in the control group. It is important to note that balance ability is closely associated with the recovery of walking ability and the probability of falling down in stroke patients.^[35]^ Furthermore, Liston and Brouwer^[36]^ reported that good postural control in balance may be closely correlated with the outcome of functional task performance during the activities of daily living. Confirming those findings, the stroke patients in our treatment group showed improvements in balance, as well as walking and self-care ability, after 3 weeks’ training. Furthermore, the BBS and TUG values before training were significantly correlated with FAC and BI, these results demonstrated that balance training via visual feedback of the Pro-kin system could strengthen the associations of walking and self-care abilities with balance. Therefore, we propose that the loss of balance function may be the main reason underlying poor walking and slower self-care abilities among stroke patients.

The fact that the improvements in balance, walking, and self-care abilities in stroke patients resulting from using the Pro-kin visual feedback system were superior to those of conventional training methods may be attributed to the distinctive properties of the Pro-kin system. The Pro-kin system, which is similar to the Biodex Balance Master^[37]^ but is based on a force platform with a regular, flat surface fixed to 4 force-transduction systems, is a comprehensive training system for human balance using visual feedback. Patients use their own sense of balance, as well as dynamic feedback on postural sway from the Pro-kin system. A variety of deep and superficial sensations and complex sensory stimuli are provided to the brain to adjust the patients’ control of their center of gravity, to enhance the function of nerve synapses, to induce cortical reorganization of nerve motor pathways,^[38]^ and also to promote the recovery of the balance and coordination functions and improve gait stability. At the same time, the system enhances the patients’ initiative, interest, and enthusiasm toward the training, thereby hastening the improvement in their balance function.

Although the present study has demonstrated the usefulness of the Pro-kin system, there were a few limitations in the study design, including a small sample size and the short duration of the training period (only 3 weeks). In addition, patients with cognitive or visual disorders, or other conditions that might affect their understanding of the task, were excluded from the selection process; therefore, further study is required to evaluate the effectiveness of the Pro-kin system in treating patients with these disorders.

## 5 Conclusions

In conclusion, these results have demonstrated that visual feedback balance training with the Pro-kin system is of significant benefit for patients after stroke, and that the use of the Pro-kin system in combination with conventional training is a viable training method for improving walking and self-care abilities of stroke patients.

## Acknowledgments

The authors thank all of the study participants, and the Spandidos Publications English Language Editing Service which provided the professional language editing service.

## Author contributions

**Conceptualization:** Min Zhang, Hong You.

**Data curation:** Min Zhang, Hongxia Zhang, Weijing Zhao, Tingting Han.

**Formal analysis:** Min Zhang, Hong You.

**Funding acquisition:** Min Zhang, Hong You.

**Investigation:** Min Zhang, Shangrong Jiang, Jia Liu.

**Methodology:** Min Zhang, Hongxia Zhang, Weijing Zhao.

**Project administration:** Min Zhang, Hongxia Zhang, Jia Liu.

**Resources:** Min Zhang, Tingting Han, Shangrong Jiang.

**Software:** Min Zhang, Hongxia Zhang, Weijing Zhao, Tingting Han, Jia Liu, Xianhui Feng.

**Supervision:** Min Zhang, Hong You, Tingting Han.

**Validation:** Min Zhang, Hong You, Hongxia Zhang, Weijing Zhao.

**Visualization:** Min Zhang, Xianhui Feng.

**Writing – original draft:** Min Zhang.

**Writing – review & editing:** Min Zhang, Hong You, Hongxia Zhang.
